# Effects of biomarkers of oxidative stress damage on prevalence and severity of visual disability among black Central Africans

**Published:** 2012-06-19

**Authors:** B. Longo-Mbenza, M. Mvitu Muaka, E. Cibanda Yokobo, I. Longo Phemba, E. Mokondjimobe, T. Gombet, D. Kibokela Ndembe, D. Tulomba Mona, S. Wayiza Masamba

**Affiliations:** 1 Faculty of Health Sciences, Walter Sisulu University, Mthatha, South Africa; 2 Department of Ophthalmology, University of Kinshasa, DR Congo; 3 School of International Studies Wendzou Medical College, China; 4 Faculty of Health Sciences, University of Marien Ngouabi, Brazzaville, Republic of Congo; 5 Department of Neuropsychiatry, University of Kinshasa, DR Congo; 6 Biostatistics Unit, Lomo Medical Center and Heart of Africa Center of Cardiology, Kinshasa, DR Congo; 7 Department of Chemistry and Chemical Technology, Walter Sisulu University, Mthatha, South Africa

## Abstract

**Background:**

Because of the demographic transition, lifestyle changes, urbanization, and nutrition transition, Central Africans are at higher risk of ocular diseases associated with oxidative stress and visual disability. This study aimed to estimate the normal values of oxidant status defined by oxidized low-density lipoprotein (Ox-LDL), 8-Isoprostane and 8-hydroxy-deoxyguanosine (8-OHdG) and to determine their pathogenic role in the prevalence and the severity of visual disability among these black Africans.

**Methods:**

This was a cross-sectional study, run in a case-control study randomly selected from Kinshasa province, DR Congo. The study included 150 type 2 diabetes mellitus (T2DM) patients (cases) matched for sex and age to 50 healthy non diabetic controls. Logistic regression models were used to identify independent determinants of visual disability.

**Results:**

The presence rates were 8.5% for blindness, 20.5% for visual impairment and 29% for visual disability including blindness and visual impairment. After adjusted for taro leaves intake, red beans intake, T2DM, aging, waist circumference, and systolic blood pressure, we identified low education level (OR=3.3 95%CI 1.5–7.2; p=0.003), rural-urban migration (OR=2.6 95% CI 1.2–5.6; p=0.017), and high Ox-LDL (OR=2.3 95% CI 1.1–4.7; p=0.029) as the important independent determinants of visual disability. After adjusted for education, intake of red beans, intake of taro leaves, triglycerides, and T2DM, we identified no intake of safou fruit (OR=50.7 95% CI 15.2–168.5; p<0.0001), rural-urban migration (OR=3.9 95%CI 1.213; p=0.012), and high 8-OHdG (OR=14.7 95% CI 3.9–54.5; p<0.0001) as the significant independent determinants of visual disability. After adjusted for education level, no intake of red beans, no intake of Taro leaves, triglycerides, and T2DM, we identified no intake of Safou fruit (OR=43.1 95% CI 13.7–135.4; p<0.0001), age≥60 years (OR=3.4 95% CI 1.3–9; p=0.024), and high 8-Isoprostane (OR=11 95% CI 3.4–36.1; p<0.0001) as the significant independent determinants of visual disability.

**Conclusions:**

Visual disability remains a public health problem in Central Africa. Antioxidant supplement, fruit intake, nutrition education, control of migration, and blocking of oxidative stress are crucial steps for delayed development of vision loss.

## Introduction

The elimination of preventable blinding diseases as a public health problem by the year 2020, is one of the three components of the World Health Organization (WHO) “VISION 2020 initiative: the Right to Sight.” According to WHO, about 284 million people in the world suffer from visual impairment [1].

Recent studies and patented new ophthalmic compositions suggest a potential prevention of visual dysfunction and a potent medicine to treat blinding ocular diseases using antioxidants in the eye [2-4].

Such specificity may be explained by the fact that diet rich in antioxidants (vitamins) and developed pro drugs prevent reactive oxygen species (ROS)-induced oxidation of lipids and proteins in the inner mitochondrial membrane in vivo and outside mitochondria in the cellular and tissue structures of the lens and eye compartments [2]. However, under pathological conditions, light exposure, aging, ROS, pro-oxidants (free radicals, atoms or clusters of atoms with a single unpaired electron determined by external and internal factors under aerobic metabolism), can increase, surpassing the body’s detoxification capacity and thus contribute to molecular level organic pathology [5]. The literature provides exhaustive catalogs of all available oxidative stress measures. Available biomarkers of oxidative stress in our context proceed by lipid peroxidation (8-isoprostane and oxidized low-density lipoprotein) and by DNA oxidation (8-hydroxy-deoxyguanosine). Oxidation of lipid molecules may result in reduced structural fluidity of these compounds with loss of integrity of cellular membrane, while oxidation of DNA molecules can result in mutation [5].

Because of epidemiologic transition (emergence of non-communicable diseases such as hypertension, metabolic syndrome, obesity, and diabetes mellitus versus decrease of infectious diseases), lifestyle changes (physical inactivity, smoking, alcohol intake), urbanization, rural-urban migration, poverty, advanced nutrition transition [6], and demographic transition (Aging), Central Africans in Kinshasa town are exposed to external sources of free radicals such as environment with ionizing radiation and pollution (heavy metals in soils and food plants, dioxins in clays; unpublished), cigarette smoke, as well as dietary intake of excessive alcohol, unsaturated fat, refined sugars, and salt, but lack or very low intake of fruits and vegetables [7]. They are also at higher risk of cardiometabolic and ocular pathologies often associated with oxidative stress and/or with visual disability [8-14].

Up to now, no published data in the Democratic Republic of Congo (DRC), Central Africa, addressed these issues. Therefore, this study aimed to estimate the normal values of oxidant status defined by oxidized low-density lipoprotein (Ox-LDL), 8-isoprostane, and 8-hydroxy-deoxyguanosine (8-OHdG), and to determine their pathogenic role in the prevalence and the severity of visual disability among these Central Africans.

## Methods

### Design, period, and setting

This was a cross-sectional, analytic and community-based survey conducted in Kinshasa town, RDC, from July to September, 2010, from a case-control study.

Kinshasa, the largest city (7 million inhabitants) enjoys a tropical climate and constitutes an Hinterland with 4 administrative districts (Mont Amba, Funa, Lukunga, Tshangu). Permission for the study was obtained from the authorities of St. Joseph Hospital in Kinshasa Limete, RDC. The study protocol was approved by the University of Kinshasa Medical Ethics Committee and the study was performed in full compliance with the Declaration of Helsinki II.

### Participants and sampling

Two groups were divided on type 2 diabetes and non-diabetic participants. A 10% simple random of adults with type 2 diabetes mellitus (T2DM, n=156 cases) was drawn from the list of all T2DM patients managed at the Ophthalmology division, St Joseph hospital, DRC, Kinshasa between May and June 2010. These cases were matched for gender and age to fifty two apparently healthy controls from Kinshasa general population; 13 participants randomly selected from each district.

Among eligible cases, six refused to participate in, while two individual eligible controls did not accept to participate in the study.

### Data collection

The structured and standardized questionnaire, administered to each participant during 30 min, sought relevant information on age, gender, rural-urban migration, residence, education level, cigarette smoking, alcohol intake, socioeconomic status (SES), ethnicity and diet.

### Dietary assessment

To assess dietary intake, a-24 H recall of eating taro leaves (*Colocasia Antiquorum* ; Yes or No) and a 10-Month recall of eating Safou fruit (*Dacryodes edulis*; Yes or No) were collected.

### Clinical measurements

Weight, height and waist circumference (WC) were measured in a standard fashion by trained, certified observers. Height was measured with a portable stadiometer and recorded to the closest 0.1 cm. WC was measured with a non stretchable tape measures to the nearest 0.1 cm. Weight was measured with a Soehnle beam scale (Soenle-Waagen Gmbh Co, Murrhardt, Germany) to the nearest 0.1 Kg. All instruments were calibrated once weekly. Blood pressure including systolic blood pressure (SBP) and diastolic blood pressure (DBP), was measured from the right arm of the seated participant after 15 min with an electronic validated digital devices (OMRON M7; Intelle/Sense, Kyoto, Japan).

### Laboratory data

Fasting (10–12 overnight fast and Post-prandial fast) blood glucose (hexokinase glucose-6-phosphate dehydrogenase reaction), triglycerides, total cholesterol, low density lipoprotein cholesterol (LDL-C), and high density lipoprotein cholesterol (HDL-C) were measured on commercially available kits (Biomerieux, Marcy l’Etoile, France) and a Hospitex autoanalyzer (Hospitex Diagnostic, Florence, Italy).

Antibodies against Oxidized LDL-cholesterol (Ox-LDL) were measured using solid phase two-side immunoassay based on the direct sandwich technique (Mercodia AB, Uppsala, Sweden). Commercial Cayman’s kits (Cayman Chemical Company, Ann Arbor, MI) were used to measure 8-Isoprostane in plasma. Serum 8-OHdG levels were measured using a competitive enzyme linked immunosorbent assay method on Biomerieux Reader version and commercial Kits supplied by Northwest Laboratories (Northwest Life Science Specialties, LLC, Vancouver, Canada).

### Eye examination

Eye examination of each participant included visual acuity measurement, ocular alignment and motility, pupil reactivity and function, visual fields, intraocular pressure, slit lamp examination of the cornea, iris, lens and vitreous, and dilated fundus examination. This fundus examination was detailed and performed at the best possible mydriasis, after dilating the pupils with tropicamide 1% and phenylephrine 10%, by indirect ophthalmoscopy at the slit lamp (Haag Steit 900, Koeniz, Switzerland) with 90 D lens.

Participants were refracted with the use of standard subjective refraction techniques. Visual acuity (VA) was measured separately for each eye and was defined according to the lowest line on the Snellen chart for which the majority of letters were read correctly, with the full required distance correction as determined by the decimal optometric scale. Best corrected VA was defined as the VA in the better eye with full distance correction.

Due to limited resources, retinal photography was excluded as a diagnostic tool in sub-Saharan Africa [15].

### Definitions

Diagnosis of T2DM was based on criteria established by the American Diabetes Association Expert Committee [16]: exhibiting either a fasting plasma glucose concentration of ≥126 mg/dl (7.0 mmol/l) on more than one occasion, and/or pharmacological treatment of diabetes.

Total obesity was defined by body mass index (weight in Kg/height in m^2^) ≥30 Kg/m^2^ [17].

Metabolic syndrome (Mets) was diagnosed according to the IDF criteria: WC≥94 cm for men, WC≥80 cm for women, triglycerides levels ≥1.7 mmol/l, HDL-cholesterol <1.03 mmol/l (male) or <1.29 mmol/l (female), SBP ≥130 mmHg or DBP ≥85 mmHg, or treatment for lipid abnormalities, and fasting plasma glucose ≥100 mg/dl (5 mmol/l) [18].

Arterial hypertension was defined as SBP ≥140 mmHg or DBP ≥90 mmHg or under antihypertensive drug treatment as recommended by the ISF/WHO guidelines Committee [19].

Aging was defined as age ≥60 years. The intake of ≥4 glasses of beer/day was considered as excessive alcohol intake. Smoking habits were classified into 2 groups: never smoker and current smoking ≥1 cigarette per day. Longer duration of diabetes was ≥5 years (median).

A diagnosis of diabetic retinopathy (DR) was made only for participants who had a minimum of one microaneurysm in any field, in addition to exhibiting hemorrhages (dot, blot, or flame shaped) and maculopathy (with and without clinically significant macula edema).

For classification of DR, the modified Airlie House classification as introduced by Early Treatment Diabetic Retinopathy Study (ETDRS) [20] was used as follows: non proliferative (NPDR), proliferative (PDR), and maculopathy.

Visual Disability (VD) was defined as blindness and visual impairment using the World Health Organization (WHO) definition and the recommendation for the revision of classification [21]. Blindness was defined as VA <0.1 (<6/60) and visual impairment (low vision) was defined as VA <0.3≥0.1 (<6/18≥6/60). Normal vision was defined by a VA between 1.0 and 0.3 (6/6–6/18).

Uncontrolled diabetes was defined by fasting plasma glucose ≥126 mg/dl at evaluation.

Self-reported ethnicity included Kongo tribe from South-West of DRC, Ngala tribe from the North-West, Luba tribe from the Centre and Swahili people from the Eastern part of DRC. According to the 4 administrative districts of Kinshasa town, the environment of residence of participants was defined as rural for Lukunga and Tshangu districts and urban for Funa and Mont Amba districts.

The education level included illiteracy, primary school, high school, and university levels. Low education level was defined as illiteracy and primary school levels. High education level included secondary school (High school) and university levels.

Socioeconomic status (SES) included low (lack of income, unemployment) and high SES.

### Statistical analysis

Qualitative data were expressed as frequency (number=n) and proportions (%). Continuous data were presented as mean±standard deviation (SD).

Comparisons of variables between two groups were performed with Student *t*-test and Chi-square test for continuous and categorical variables, respectively. Comparisons of variables between three groups were performed with one way ANOVA (ANOVA) and Bonferroni Post-Hoc test for continuous variables, while P for trend test was used in comparisons of proportions of qualitative variables.

The univariate risk of visual disability was assessed in calculating Odds ratio (OR) with 95% confidence intervals (95% CI). Multivariate analyses such as logistic regression models, were used to assess the independent effect of each biomarker of oxidative stress on the presence of visual disability after adjusting for the effect of confounding factors and avoiding collinearity in each logistic regression model.

The receiver operating characteristic curve for Ox-LDL, 8-Isoprostane, and 8-OHdG to predict the presence of VD, was plotted. The optimal cut off point of biomarkers were calculated by plotting sensitivity against 1-specificity.

A p-value <0.05 was considered significant.

Data analysis was performed using the Statistical Package for Social Sciences (SPSS) for Windows version 19 (SPSS Inc., Chicago, IL).

## Results

A total of 200 participants (response rate of 96.2% in type 2 diabetics and response rate of 96.2% in controls) including 90 males and 110 females (sex ratio of 1:1) were examined.

The prevalence rates were 8.5% for blindness (n=17), 20.5% for visual impairment (n=41) and 29% for visual disability (n=58).

**Figure 1:**
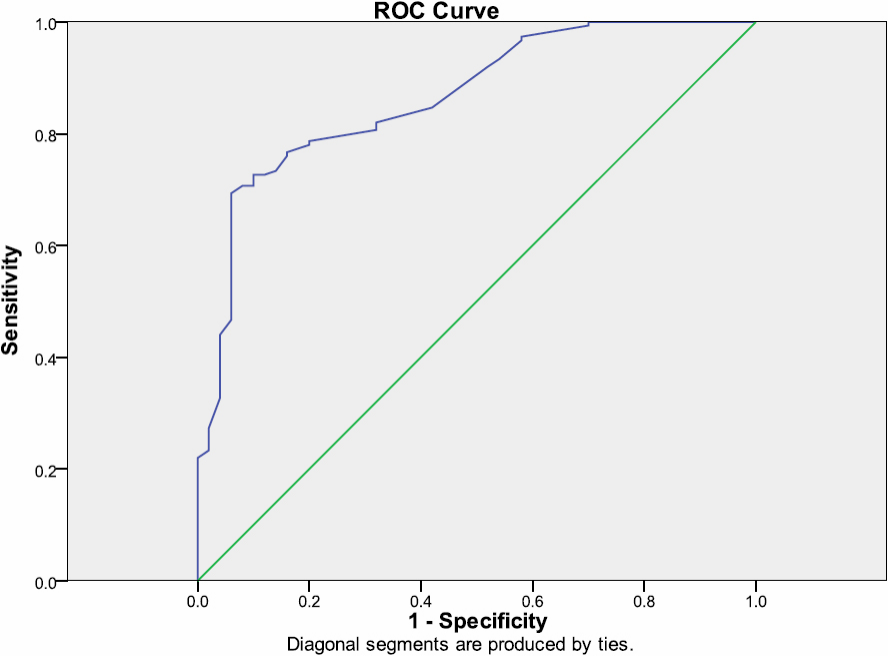
ROC curves for 8 hydroxydeoxyguanosine (8-OHdG) to discriminate T2DM patients from non diabetic controls; AUC=0.865 95%CI 0.809–0.920; p<0.0001.

**Figure 2:**
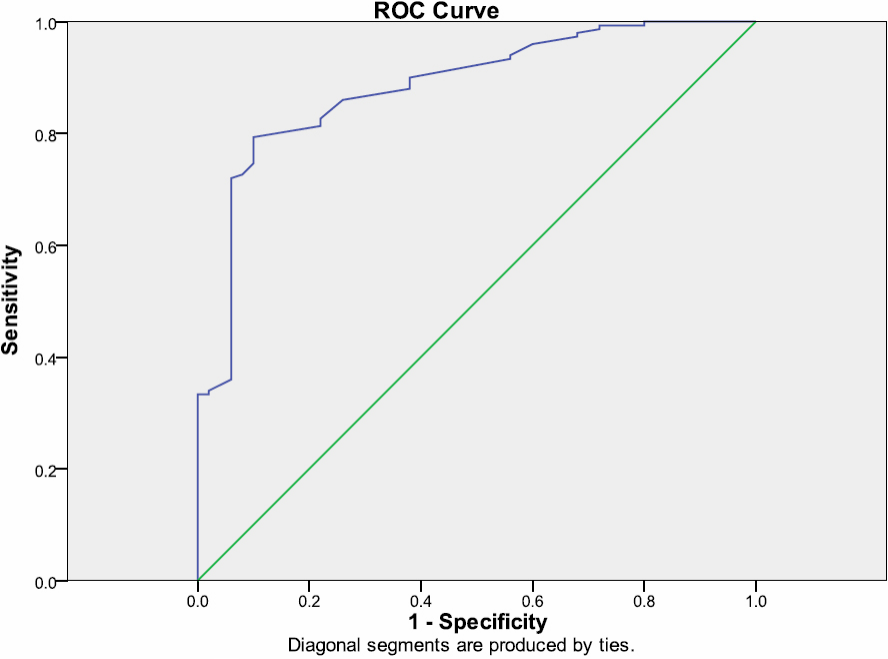
ROC curves for 8-isoprostane to discriminate T2DM patients from non diabetic controls; AUC=0.884 95%CI 0.831–0.936; p<0.0001.

The mean values of continuous variables of participants according to the presence of visual disability are shown in Table 1. The mean values of WC, SBP, blood glucose, 8-isoprostane, and 8-OH-dG were significantly higher in people with visual disability than participants without visual disability. Figure 1 and Figure 2 showed the ROC curves that served to calculate AUC and elevated cut-offs for 8-isoprostane level>55 ng/ml (sensitivity=78% and specificity=100%) and 8-OH-dG level>40 mg/ml (sensitivity=76% and specificity=100%).

**Table 1:** Comparisons of continuous variables according to Visual Disability (VD) in all participants.

**Variables of interest**	**Presence of VD mean±SD**	**Absence of VD mean±SD**	**p-value**
Age (years)	56.7±15.3	53.3±11.8	0.086
Weight (Kg)	67.2±11.1	66.4±14.1	0.687
Height (m)	1.643±0.074	1.659±0.474	0.796
BMI (Kg/m^2^)	24.9±4.4	25±5	0.972
WC (cm)	94.8±16.6	90±14.6	0.040
SBP (mmHg)	131.4±24.2	124.3±19.6	0.032
DBP (mmHg)	79.5±12.8	77.8±12.2	0.389
Ox-LDL (U/L)	62.6±18.6	56.8±20.8	0.67
PG in post prandial for non diabetics or FPG in diabetics (mg/dl)	186.1±82.8	158±73.7	0.019
8-isoprostane (ng/ml)	101.3±51.4	58.8±32.3	<0.0001
8-OHdG (mg/ml)	73.7±27.8	46.3±21.8	<0.0001
Total cholesterol (mg/dl)	188.3±48.1	198.3±56.9	0.238
Triglycerides (mg/dl)	125.8±46.4	114.2±46.5	0.110
LDL-C (mg/dl)	81.3±41	78.5±45.3	0.675
HDL-C (mg/dl)	51.5±14.7	53.9±15.5	0.312

Age ≥60 years, migration, low education level (Table 2), as well as no intake of safou fruit, presence of T2DM, elevated Ox-LDL, elevated 8-Isoprostane, and elevated 8-OH-dG had individual positive and significant effect on development of visual disability.

**Table 2:** Univariate risk of visual disability (VD) in all participants: role of gender, age, smoking, excessive alcohol intake, education level, and migration.

** **	**Presence of**		** **
**Variables of interest**	**n (%)**	**OR (95%CI)**	**p-value**
**Gender**			
Males	31 (34.4)	-	** **
Females	31 (28.2)	-	0.341
**Aging**			
≥60 years	28 (36.8)	1.8 (1.01–3.4)	0.040
<60 years	30 (24.2)	** **	** **
**Smoking**			
Yes	5 (45.5)	-	0.180
No	56 (29.8)	** **	** **
**Excessive alcohol intake**			
Yes	17 (35.4)	-	0.448
No	45 (29.6)	** **	** **
**Education level**			
Low	43 (33.6)	1.9 (1.02–3.8)	0.039
High	15 (20.8)	** **	** **
**Migration**			
Yes	46 (34.6)	2.4 (1.2–5)	0.014
No	12 (17.9)	** **	** **

**Figure 3:**
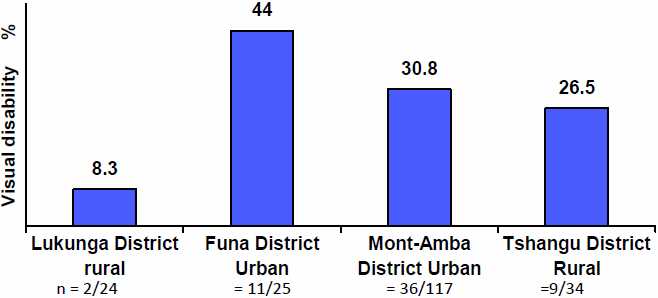
Distribution of visual disability rates in all participants and across Kinshasa Districts.

Figure 3 shows a significant (p=0.046) and unequal distribution of visual disability rates across Kinshasa districts; the highest rates observed in the two urban districts of Funa and Mont Amba gave a sum of 37.4% versus 19% for rural districts of Lukunga and Tshangu.

Univariate risk factors of visual disability such as no intake of safou, taro leaves and red beans, total obesity, abdominal obesity, T2DM, elevated Ox-LDL, elevated 8-isoprostane, and high 8-OHdG are shown in Table 3.

**Table 3:** Univariate risk of visual visability (VD) in all participants conferred by dietary intake, cardiometabolic conditions and elevated levels of biomarkers of oxidative stress.

** **	**Presence of VD**		** **
**Variables of interest**	**n (%)**	**OR (95% CI)**	**p-value**
**Safou fruit intake**			
No	40 (88.9)	60.9 (21.3–174.3)	<0.0001
Yes	18 (11.6)	** **	** **
**Taro leaves (*Colocasia antiquorum*)**			
No	47 (31.3)	-	0.860
Yes	15 (30.0)	** **	** **
**Red beans intake**			
No	23 (35.4)	-	0.352
Yes	39 (28.9)	** **	** **
**Total obesity**			
Yes	37 (29.1)	-	0.956
No	21 (28.8)	** **	** **
**Abdominal obesity**			
Yes	30 (32.6)	-	0.299
No	28 (25.9)	** **	** **
**T2DM**			
Yes	53 (35.3)	4.9 (1.8–13.1)	<0.0001
No	5 (10.0)	** **	** **
**Elevated Ox-LDL**			
Yes	44 (34.6)	2.2 (1.1–4.4)	0.020
No	14 (19.2)	** **	** **
**Elevated 8-isoprostane**			
Yes	47 (40.2)	4.4 (2.1–9.2)	<0.0001
No	11 (13.3)	** **	** **
**Elevated 8-OHdG**			
Yes	48 (39.3)	4.4 (2.1–9.4)	<0.0001

After adjusting for taro leaves (*Colocasia antiquorum*) intake, red beans intake, T2DM, aging, WC, and SBP, the regression logistic model 1 identified low education level, migration, and elevated Ox-LDL as the independent and significant determinants of visual disability in this population (Table 4).

**Table 4:** Independent deleterious role of low education, migration and elevated oxLDL on visual disability (VD) in Central Africans.

**Independent variables**	**B coefficient**	**Standard error**	**Wald χ^2^**	**OR (95% CI)**	**p-value**
**Education level**					
Low versus High	1.192	0.400	8.877	3.3 (1.5–7.2)	0.003
**Migration**					
Yes versus No	0.945	0.395	5.724	2.6 (1.2 – 5.6)	0.017
**Oxidized LDL-C**					
Elevated versus Normal level	0.820	0.375	4.788	2.3 (1.1 – 4.7)	0.029
Constant	-	3.049	0.555	30.174	<0.0001

Table 5 shows that no intake of safou fruits, age ≥60 years, and elevated 8-isoprostane were the independent determinants of visual disability in the population when the logistic regression model 2 was adjusted for education level, intake of red beans, intake of taro leaves, triglycerides, and T2DM.

**Table 5:** Independent role of 8-isoprostane on the presence of visual disability in Central Africans.

**Independent variables**	**B coefficient**	**Standard error**	**Wald χ^2^**	**OR (95% CI)**	**p-value**
**Safou fruit intake**					
No versus Yes	3.764	0.584	41.610	43.1 (13.7–135.4)	<0.0001
**Migration**					
Yes versus No	1.348	0.598	5.083	3.9 (1.2–12.4)	0.024
**Aging**					
≥60 years versus <60 years	1.225	0.496	6.100	3.4 (1.3–9)	0.014
**8-isoprostane**					
High versus Normal level	2.397	0.607	15.609	11 (3.4–36.1)	<0.0001
Constant	-	5.413	0.900	36.163	<0.0001

No intake of safou fruits, migration, age ≥60 years and elevated 8-OH-dG were significantly, positively, and independently associated with the presence of visual disability in the study after adjusting for education level, intake of red beans, intake of taro leaves, triglycerides, and T2DM (Table 6).

**Table 6:** Independent role of 8-OHd guanosine on the presence of visual disability in Central Africans.

**Independent variables**	**B coefficient**	**Standard error**	**Wald χ^2^**	**OR (95% CI)**	**p-value**
**Safou fruit intake**					
No versus Yes	3.925	0.613	41.004	50.7 (15.2–168.5)	<0.0001
**Migration**					
Yes versus No	1.358	0.616	4.849	3.9 (1.2–13)	0.028
**Aging**					
≥60 years versus <60 years	1.247	0.499	6.255	3.5 (1.3–9.3)	0.012
**8-OHd guanosine**					
High versus Normal level	2.685	0.670	16.032	14.7 (3.9–54.5)	<0.0001
Constant	-	5.774	0.985	34.332	<0.0001

**Figure 4:**
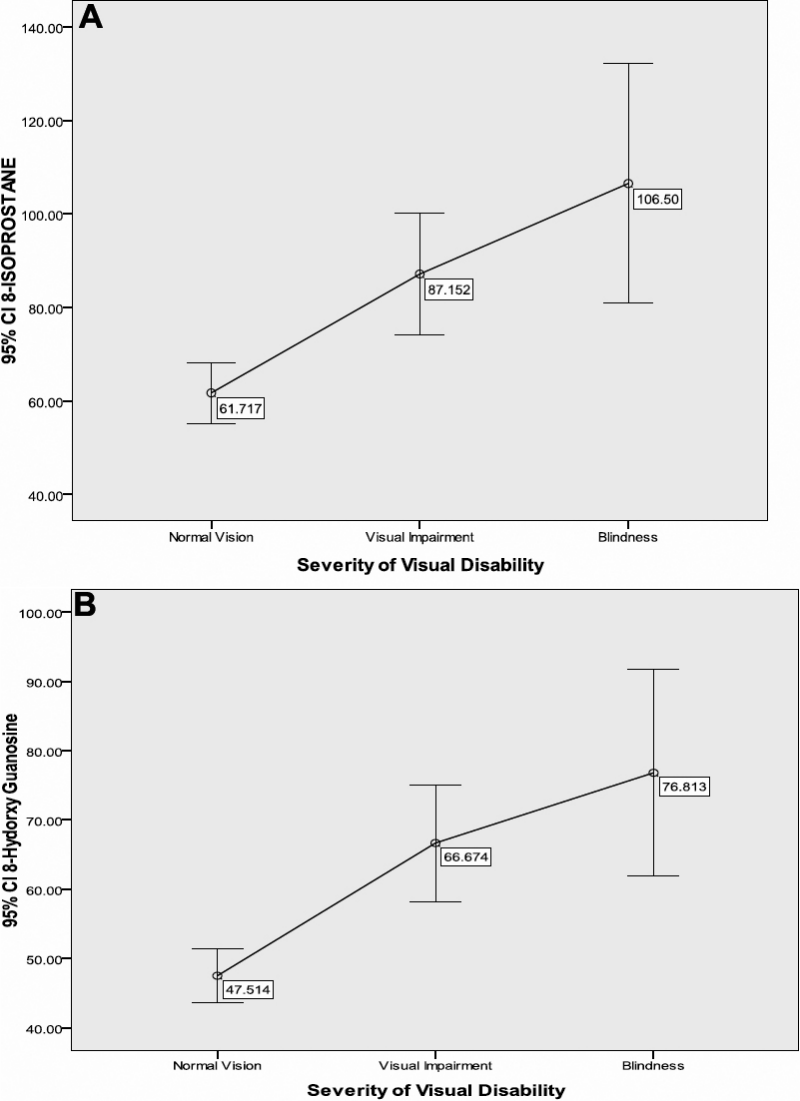
Relationship between biomarkers of oxidative stress and visual disability.

There was a significant, dose–response and linear relationship between significant biomarkers of oxidative stress (8-isoprostane and 8-OH-dG in each y-axis) and severity of visual disability(x-axis): increase in their values from normal vision toward visual impairment and blindness (Figure 4).

## Discussion

The present survey was conducted in a population at higher risk of chronic diseases. To our knowledge, this is the first African study to assess the levels of Biomarkers using ROCs and their independent role in the development of visual disability.

The current burden of visual disability in both non-diabetic and diabetic participants and its severity from visual impairment to blindness, was consistent with our previous paper [22], studies from Cameroon [23] and from Jordan [24], but higher than that reported from DRC in 1994 [25] and 2009 [12]. This burden may be explained by nutrition transition [6], explosing T2 DM [7], political crisis-induced poverty, aging, lifestyle changes and oxidative stress.

In univariate analysis, higher levels of WC, SBP, ox-LDL, blood glucose, 8-isoprostane, and 8-OH-dG, as well as no intake of safou fruit, age ≥60 years, urban residence, low education level, and migration were identified as the factors significantly and positively associated with the presence of visual disability among these Congolese. Gender and the rest of variables were not associated with visual disability.

However, after avoiding collinearity and confounding factors such as T2DM, we identified age ≥60 years, migration, low education level, no intake of safou fruit, and higher oxidant status as independent determinants of the presence of visual disability in these Central Africans. This suggests a synergistic, isolated or interactive action of non modified factors (aging), endogenous attributes of people, and environmental factors in the development of visual disability as observed in multifactorial and chronic diseases.

In healthy individuals, the eye needs specific nutrients such as safou fruit rich in antioxidants and vitamins [26,27]. In Kinshasa town, fruits are very expensive in general and during seasons of fruits in particular. No intake of safou, a season fruit, conferred the highest risk of the development of visual disability because of possible inefficient antioxidant status.

Aging, however, reduces the levels of these nutrients, and the eyes’ capability to remove free radicals. The essential antioxidants within safou fruit may keep ocular tissues healthy.

After rural-urban migration with settlement in urban residence, the eye is exposed to Kinshasa environment with pollution, lack of traditional diet rich in vegetables, fibers and fruits. Kinshasa town is characterized by few green spaces, but longer exposure to sunlight.

Low education level may reflect lack of education on nutrition, poverty limiting access to health care for T2DM, and cultural beliefs. In DRC, green and crude vegetables in general and cabbages in particular are considered foods for goats, while fruits are left for children.

The demographic, environmental, and dietary factors identified in this study may facilitate the initiation and the acceleration of imbalance of oxidant/antioxidant status and visual disability. Indeed, there was an increase in levels of oxidant status (elevated 8-Isoprostane and elevated 8-OH-dG) with the presence and the severity (progression) of visual disability in these Congolese.

Increase in OxLDL and 8-isoprostane is determined by lipid peroxidation which occurs in response to elevated levels of free radicals. Chronic hyperglycemia in diabetics and acute hyperglycemia induced by post-prandial time may be responsible for the elevated OxLDL and 8-Isoprostane in these Congolese with visual disability.

Oxidation of LDL is a complex process taking place in both the extra- and intra-cellular space. Numerous studies have clearly acknowledged that the levels of circulating plasma of Ox-LDL are associated with obesity-related metabolic disturbance such as metabolic syndrome and diabetes mellitus [28]. In this study, both high levels of WC, SBP, and blood glucose, components of metabolic syndrome, as well as T2DM were associated with the presence of visual disability in univariate analysis, while elevated oxLDL was associated with the presence of visual disability in multivariate analysis.

The present study showed that visual disability was associated with increased DNA damage defined by high levels of 8-OH-dG. Aldebasi et al. [29] indicate significantly higher concentrations of serum 8-OH-dG in Saudi type 2 diabetic patients with retinopathy compared to type 2 diabetic patients without retinopathy. Diabetic retinopathy, one of the leading causes of visual disability worldwide among adults and associated with oxidative stress [29], may be involved in the development of observed visual disability.

The present study showed that elevated levels of Ox-LDL, 8-isoprostane, and 8-OH-dG, identified as independent determinants of the presence of visual disability, were also significantly associated with the progression and severity of vision loss. It illustrated that the levels of Ox-LDL, 8-Isoprostane, and 8-OHdG in participants with blindness were higher than those in participants with visual impairment and participants with normal vision, respectively. The levels of these biomarkers of oxidative stress in participants with visual impairment were also higher than those in people with normal vision.

### Clinical implications and perspectives for public health

The sensitive and specific levels of 8-isoprostane and 8-OH-dG will be used in different diseases related to oxidative stress. The present findings will impact on nutrition education, integrating ophthalmic practice into Primary Health care systems, and the WHO “VISION 2020 initiative” in Central Africa. Training ophthalmologists with skills in clinical nutrition, infrastructures for early diagnosis of visual disability and recommended diet rich in antioxidants are needed to eliminate preventable blinding diseases as public health problems. This calls for the implementation of more preventive strategies among this population with low education level, low intake of fruits and vegetables, facing urban inequalities after rural-urban migration, nutrition transition and aging.

The current management strategy for vision loss requires early detection of nutrients deficiency and oxidative stress and optimal control of WC, blood pressure, and blood glucose to slow the progression of visual dysfunction.

Most of these Central Africans require early and good binocular visual acuity screening. These results suggest the need of drugs for treating several oxidative stress-related ocular diseases of aging eye, protecting against vitamins deficiency at early stages of ocular diseases, and delaying the progression of vision loss [2-4].

### Study limitations

This study may be limited to some degree because of its cross-sectional design which is not able to demonstrate a causal association between the identified independent determinants and visual disability presence and progression.

### Conclusion

These findings emphasize the important and independent role of aging, no intake of safou fruit, rural-urban migration, low education level, and oxidative stress in the presence and the progression of visual disability.

Antioxidant supplements, nutrition education, control of migration, and blocking of oxidative stress are crucial steps for delayed onset, progression and severity of visual disability in general and in aging people in particular.
